# Active Haptic Exploration of Softness: Indentation Force Is Systematically Related to Prediction, Sensation and Motivation

**DOI:** 10.3389/fnint.2018.00059

**Published:** 2018-11-29

**Authors:** Alexandra Lezkan, Anna Metzger, Knut Drewing

**Affiliations:** Department of General Psychology, Justus-Liebig University Giessen, Giessen, Germany

**Keywords:** haptic perception, softness, exploratory movements, movement control, action and perception

## Abstract

Active finger movements play a crucial role in natural haptic perception. For the perception of different haptic properties people use different well-chosen movement schemes (Lederman and Klatzky, 1987). The haptic property of softness is stereotypically judged by repeatedly pressing one’s finger against an objects’ surface, actively indenting the object. It has been shown that people adjust the peak indentation forces of their pressing movements to the expected stimulus’ softness in order to improve perception (Kaim and Drewing, 2011). Here, we aim to clarify the mechanisms underlying such adjustments. We disentangle how people modulate executed peak indentation forces depending on predictive vs. sensory signals to softness, and investigate the influence of the participants’ motivational state on movement adjustments. In Experiment 1, participants performed a two alternative forced-choice (2AFC) softness discrimination task for stimulus pairs from one of four softness categories. We manipulated the predictability of the softness category. Either all stimuli of the same category were presented in a blocked fashion, which allowed predicting the softness category of the upcoming pair (predictive signals high), or stimuli from different categories were randomly intermixed, which made prediction impossible (predictive signals low). Sensory signals to softness category of the two stimuli in a pair are gathered during exploration. We contrasted the first indentation (sensory signals low) and last indentation (sensory signals high) in order to examine the effect of sensory signals. The results demonstrate that participants systematically apply lower forces when softer objects (as compared to harder objects) are indicated by predictive signals. Notably, sensory signals seemed to be not as relevant as predictive signals. However, in Experiment 2, we manipulated participant motivation by introducing rewards for good performance, and showed that the use of sensory information for movement adjustments can be fostered by high motivation. Overall, the present study demonstrates that exploratory movements are adjusted to the actual perceptual situation and that in the process of fine-tuning, closed- and open-loop mechanisms interact, with varying contributions depending on the observer’s motivation.

## Introduction

Hand movements are a fundamental part of haptic perception. In a natural exploration process, haptic sensations are generated by active hand movements (Gibson, 1962). The way people naturally move their hands depends on what object property they are interested in (Lederman and Klatzky, 1987). Imagine two possible situations: first, you want to test how ripe a mango is. Second, you want to know whether a blouse is made out of silk. In order to explore in these two situations you would probably apply two fundamentally different movements. The ripeness of a mango is probably best judged by its softness. Softness is a central dimension in haptic perception (Bergmann Tiest and Kappers, 2006) and refers to the subjective impression of how compressible and deformable an object is. In order to explore softness people typically perform a specific movement scheme: they apply a normal force to the surface with their fingers, indent the object (Lederman and Klatzky, 1987). This movement scheme is systematically used only for softness perception and *not* for the exploration of other object properties. However, people do not only choose appropriate movement schemes, they also seem to adjust individual movement parameters to the perceptual situation. In the case of softness, it was found that higher peak forces were used for the first indentation when stimuli were expected to be hard as compared to soft (Kaim and Drewing, 2011). Here, we aim to clarify the mechanisms underlying such fine-tuning. We study whether indentation forces are systematically adjusted to gradually varying softness values, and in particular, we investigate the contribution of predictive signals, sensory signals and motivation to the fine-tuning of force over the course of a natural exploration.

Previous research found that movement parameters are adjusted in haptic exploration for various tasks. Specific movement parameters, like force, velocity, or direction, matter for different tasks. Tanaka et al. (2014), for instance, reported that participants vary their normal force, scanning velocity and break times depending on the roughness of objects. Some other studies also described how movement adjustments may influence perception (e.g., O’Malley and Goldfarb, 2002; Di Luca, 2011; Drewing, 2012). With regard to softness perception, it was reported that (especially for hard stimuli) higher indentation forces can improve softness discrimination (Srinivasan and LaMotte, 1995; Kaim and Drewing, 2011). Kaim and Drewing (2011) described a corresponding fine-tuning of the peak force in the first indentation for a discrimination task. Two interesting results were reported. First, participants exerted a higher peak force in the first indentation in a trial, when they predicted that the stimulus pair would be hard in contrast to soft. Without predictions, no difference was found between the initial peak force used for hard and soft stimuli. Second, in an additional experiment participants were instructed to indent with either less or more force than they used spontaneously. When participants indented hard stimuli with *less* force, their ability to discriminate them diminished, whereas *more* force did not improve discrimination performance. Taken together, this study suggests that people adjust indentation force based on predictive signals, and that these adjustments can improve softness perception.

The reported indentation force adjustments were only analyzed for two categories of softness. Additionally, only the first indentation was investigated, and a role for sensory information in force adjustments was neglected. However, natural exploration goes far beyond the previously investigated first indentation. People tend to repeat movements, because a single touch seems not to generate sufficient sensory signals to reach a decision (Klatzky and Lederman, 1999). This means that in natural exploration, sensory information about the stimulus is accumulated and may also be used for further movement control. Here, we aim to consider the entire process of softness exploration and the determinants of force adjustments over the course of the exploration. Therefore, we test whether indentation force adjustments occur not only based on prediction (predictive signals) but also based on sensation (sensory signals).

When the entire exploration process was considered previously, exploration movements were reported to change systematically over the course of the exploration. Lederman and Klatzky (1990) showed a corresponding two-stage exploration in haptic object identification. First, participants applied general exploration procedures, like grasping and lifting, which allowed them to obtain some initial (yet imprecise) information about a variety of properties. Second, a specific exploratory procedure was used- typically one that was associated with a property that is highly informative for the explored object. Thus, people qualitatively adjusted their exploration behavior in the second stage based on previously gathered sensory information. Some studies also reported adjustments of individual movement parameters, for instance, of speed and force to an unpredictable surface curvature (Weiss and Flanders, 2011). Another example is the study of Saig et al. (2012), in which the authors reported online adjustments of specific movement parameters for a localizing task with artificial whiskers. The authors showed that, rather than repeating the same movements all over again, movement parameters converged during the task to a steady state. Training on the task resulted in changes of the hand velocity, which was connected to better performance. This is to say, sensory signals gathered during the exploration led to adjustments of motor variables. Our study investigates whether in the natural exploration of softness a similar closed sensorimotor control loop is involved, in which—besides predictive signals—sensory signals also affect movement parameters.

That is to say, we speculate that movement parameters are influenced by feedforward processes via predictive signals; and by feedback processes via sensory signals (see Wolpert, 1997). Predictive signals are available before any interaction with the object and are, for instance, based on previous experiences or on vision. Sensory signals are gathered during the exploration. Therefore, later in the exploration process, more sensory signals are available (Lezkan and Drewing, 2018). We propose that the sensory signals from initial movements are taken into account as feedback and, thus, that they influence upcoming motor commands, which will generate further sensory signals until a decision on the to-be-judged property is reached (i.e., softness). Consequently, later in the exploration process, movements are assumed to be better tuned to fit object characteristics (here called “online adjustments”) based on sensory signals. This is similar to ideas of “Iterative Learning Control” incorporated in control theory, where repetitiveness is used for control optimization (Chen et al., 2012). Note also that the acquisition of sensory signals may vary substantially, as people are able to decide how many movements they perform. The more exploratory movements are performed, the more sensory signals are generated, and the more reliable the sensation will be (e.g., Quick, 1974; Gescheider et al., 1999; Lezkan and Drewing, 2018). Although additional exploratory movements can add sensory signals, they are also associated with additional movement costs. In consequence, there is a trade-off between benefits of additional movements (more reliable sensation) and their movement costs (effort). It has been suggested that the rewarding nature of the performed movement determines where the balance between movement costs, in terms of effort, and benefits from additional movements, in terms of additional sensory signals, is found (Todorov and Jordan, 2002; Todorov, 2004). The rewarding nature of a task can be also rephrased as the motivation to perform the task (Beckmann and Heckhausen, 2006). So far most of the evidence for the fact that higher energetic effort is spent when the task motivation (i.e., its’ rewarding nature) is higher comes from studies on eye movements (Takikawa et al., 2002; Xu-Wilson et al., 2009; Schütz et al., 2012). We assume that in free haptic exploration, motivation influences the participant’s effort, and thus the gathering and subsequent influence of sensory signals, in a similar way. Higher motivation should lead to the willingness to spent more effort in order to generate more sensory signals. Therefore, we suggest that increased motivation will lead to an increased impact of sensory signals. In particular, Experiment 1 investigates whether and in how far sensory and predictive signals lead to the adjustments of indentation force. Experiment 2 tests if effects of sensory signals are moderated by motivation.

## Experiment 1

On every trial, two deformable silicone stimuli were discriminated according to their softness. Stimuli were defined by the physical correlate of softness, namely compliance, which is the relationship between a physical force applied to an object and the resulting deformation of the object’s surface. We used four different softness categories (“soft” ~0.7 mm/N, “medium soft” ~0.4 mm/N, “medium hard” ~0.2 mm/N or “hard” ~0.1 mm/N). Both stimuli of each pair were from the same softness category and differed only by 15%–20% in their exact compliance.

We studied the influences of predictive signals on movement control by manipulating the presentation order. Hence, within one experimental block, all stimulus pairs could either be from the same softness category, which implicitly induced prior knowledge of the softness category of the upcoming stimulus pair (predictive signals high) or from all four categories (predictive signals low). Extending Kaim and Drewing (2011), we used more than two categories of softness, and tested whether movement adjustments systematically follow the softness category. We expected systematic adjustments of force with respect to object softness: peak forces should be systematically adjusted towards higher forces when it can be predicted that stimuli will be harder. Specifically, when subtracting peak forces based on low predictive signals from peak forces based on high predictive signals, we expected linearly increasing values with harder categories.

We further investigated force adjustments based on sensory signals. We focused on the first (sensory signals low) and the last (sensory signals high) indentations during each trial, because those indentations represent the two extremes of the availability of sensory signals. We expected that indentation forces would be systematically adjusted between the first and the last indentation, which would correspond to the feedback influence of sensory signals (low vs. high sensory signals, respectively). Specifically, difference values, produced by subtracting peak forces based on low sensory signals from peak forces based on high sensory signals, should show a systematic increase for less soft categories.

### Materials and Methods

#### Participants

The sample consisted of one left-handed and 15 right-handed participants (mean age: 24.9 years, range: 19–33 years; eight females). Participants from both experiments were naïve to the purpose of the experiment and were reimbursed for participating. All participants had no sensory or motor impairments or recent injuries of the right index finger, and had a two-point discrimination threshold of 2 mm or less for the right index finger. This study was carried out in accordance with the recommendations for ethical standards of the 2008 Declaration of Helsinki with written informed consent from all subjects. All subjects gave written informed consent in accordance with the Declaration of Helsinki. The protocol was approved by the local ethics committee (LEK) of FB 06 at Giessen University (approval number: 2013-0021).

#### Apparatus and Stimuli

**Figure 1:**
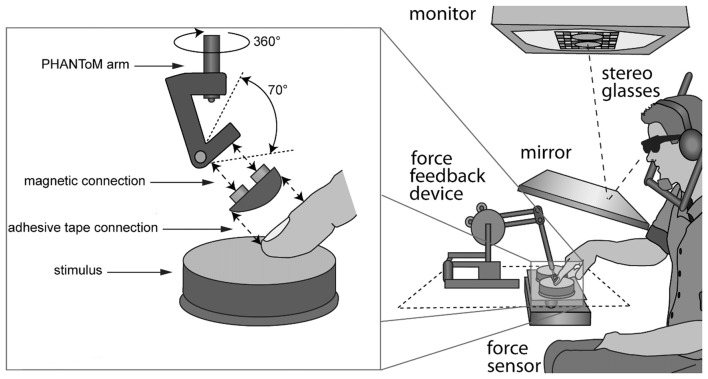
Setup. Stimuli were visually represented on a monitor and seen through a mirror and stereo glasses. Rubber stimuli were placed on a force sensor next to each other. The right index finger was connected to the PHANToM via an adapter.

Participants sat in front of a custom-made visuo-haptic workbench (Figure 1), which comprised a PHANToM 1.5A haptic force feedback device, a force sensor (682 Hz, resolution: 0.05 N), and a 22″ computer screen (120 Hz, 1024 × 1280 pixels). A head and chin rest limited head movement. Participants saw the screen via stereo glasses and a mirror, which prevented them from seeing their hand or the stimuli. The right index finger was connected to the PHANToM via an adapter for the fingernail (Figure 1). In order to make kinesthetic and tactile signals available, we used double-sided adhesive tape and an adapter, which left the finger pad bare. The PHANToM measured finger positions. The force sensor, consisting of a measuring beam (LCB 130) and a force amplifier (GSV-2AS) was placed below the stimuli. Custom software controlled the experiment, collected responses, and recorded force and position data at recording intervals of 3 ms.

**Figure 2:**
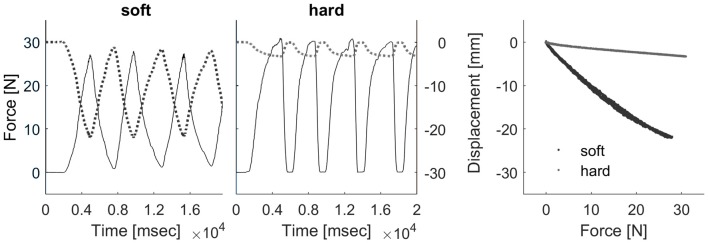
Deformation behavior of example “soft” and “hard” stimuli with “standard finger” exploration up to 30 N. The curves for both categories show that all stimuli in our range would not reach full compression for typical peak forces (Kaim and Drewing, 2011).

Participants touched two real compliance stimuli placed side by side in front of them. We produced custom-made silicone rubber discs (diameter: 75 mm; height: 38 mm) by mixing a two-component silicone rubber mold material (Alpa Sil EH 10:1) with adjusted amounts of silicone oil (polydimethylsiloxane). The final stimulus set contained compliances between 0.12 mm/N and 0.88 mm/N. Compliances were defined as the slope of the regression line, fitted to vertical surface displacement produced by a mechanical “standard finger” for forces between 0 N and 9 N. The “standard finger” was a flat-ended cylindrical probe (1 cm^2^ area—for details on compliance measurement, Kaim and Drewing, 2011). Figure 2 shows deformation of a stimulus from the “soft” and the “hard” category for “standard finger” exploration with peaks up to 30 N, which is the average range of peak forces in natural exploration. As it can be seen in the figure, the deformation of stimuli from both extreme categories (“soft” and “hard”) does not saturate within this range, which excludes full compression as a possible discrimination clue.

Stimuli were grouped into four softness categories. Each category consisted of a standard stimulus and two comparison stimuli: “hard” (standard (s): 0.14 mm/N, comparisons (c): 0.12 mm/N and 0.15 mm/N), “medium hard” (s: 0.21 mm/N, c: 0.18 mm/N and 0.24 mm/N), “medium soft” (s: 0.37 mm/N, c: 0.29 mm/N and 0.46 mm/N), or “soft” (s: 0.74 mm/N, c: 0.62 mm/N and 0.88 mm/N). The compliance differences between the stimuli of a stimulus pair were at least three times smaller than compliance differences between stimuli of different categories. The comparisons of each compliance category were chosen to differ approximately by one just noticeable difference (JND) from the standard. The calculations were based on interpolations from Weber fractions for harder (21.2%) and softer (13.5%) stimuli reported in Kaim and Drewing (2011).

Stimuli were displayed on the screen as three-dimensional (3D) cylindrical discs in a virtual 3D scene. Position and size of the “visual” stimuli corresponded to those of the real objects. Outside the stimulus area, the current finger position was visible as a sphere (8 mm diameter). No visual feedback about stimulus compliance was provided; the finger representation disappeared when the stimulus was touched (>0.1 N force).

#### Design and Procedure

In each trial, a stimulus pair, which comprised a standard and a comparison stimulus, was explored. We manipulated two within-participant variables: softness category of the stimulus pair (hard, medium hard, medium soft, soft) and presentation order (predictive signals high vs. low) of pairs within one block. For the manipulation of presentation order either all stimulus pairs of a block were taken from the same softness category (blocked condition: predictive signals high), or from all four categories (random condition: predictive signals low). In each block of the blocked condition, only the two pairs from the same softness category were presented. Therefore, a prediction of the softness category of the upcoming stimulus pair was possible (predictive signals high). In each block of the random condition, all eight stimulus pairs were presented. Therefore, no prediction of the softness category of the upcoming stimulus pair was possible (predictive signals low). We analyzed data from two Exploration Moments (first indentation: sensory signals low vs. last indentation: sensory signals high).

The experiment consisted of four sessions. Each session comprised four blocks of 96 trials (1,536 trials in total) and was conducted on a different day per session, either only blocks from the blocked, or from the random condition, were presented. After balancing which stimulus was left (standard or comparison), each of the four combinations (stimulus pair × positioning) was randomly repeated 24 times in a block of the blocked condition. For the random blocks, we balanced the positioning of the standard, and made sure that each combination was repeated six times in a block. Additionally, we balanced between participants which condition they started with in the first session. In the following sessions, the two conditions alternated from session to session. The order of softness categories in a blocked session was counterbalanced across participants according to a Latin square and stayed the same for the two blocked sessions of one participant.

On each trial, participants performed a two alternative forced-choice (2AFC) discrimination task, judging which stimulus was softer. A cross indicated the center of the stimulus to be touched. Only the left or the right stimulus was presented on the screen before the first touch, which indicated which stimulus to explore first. Participants were free to perform as many indentations as they wanted, and to switch between left and right stimuli at any point in time. No immediate feedback about the correctness of the answer was given. However, at the end of each session, the percentage of correct trials was shown, so that participants would be motivated to perform equally well in all sessions.

#### Data Analysis

**Figure 3:**
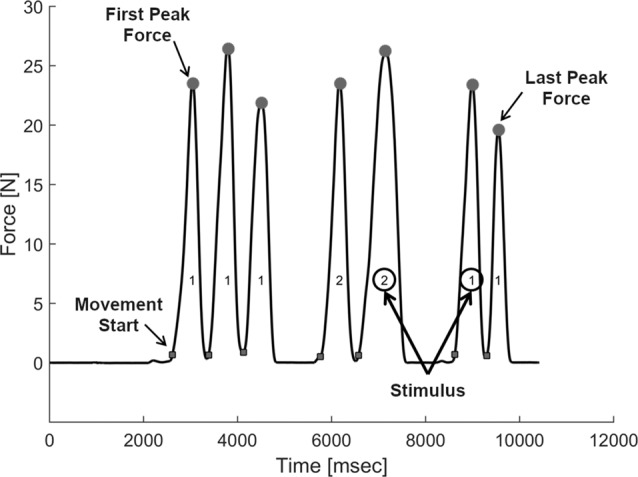
Example trial for exploration of one stimulus pair. As it can be seen, participants were free to indent the stimuli and switch between the two stimuli of a pair, as often as they wished. The difference between the last first peak indentation force is the measure of the effect of sensory signals.

We analyzed the first and last indentations performed on the stimulus pair for each trial (Figure 3). We focused on the peak indentation forces, which play an important role in softness perception (Srinivasan and LaMotte, 1995; Tan et al., 1995). Peak forces were defined as the forces for which the derivative of force over time changed from positive to negative. Force signals were previously smoothed by a moving-averaging window with a kernel of 45 ms. We restricted the time interval between two peaks to be at least 180 ms in order to exclude finger shaking or movement pauses. We calculated average individual peak forces for the first and last indentations per experimental condition (Presentation Order × Softness Category). To test for systematic effects, we used a linear contrast analysis of differences produced by predictive signals (predictive signals low vs. predictive signals high) as well as those produced by sensory signals (sensory signals low vs. sensory signals high). This is to say, we calculated for each softness category the difference in peak forces between blocked and random sessions (effect of predictive signals) as well as between the first and last indentations (effect of sensory signals). Those difference values were tested in linear contrast analyses, in which the linear combination of mean values for softness category is tested against “0.” We expected that difference values produced by the effect sensory signals as well as those produced by the effect of sensory signals systematically increase for less soft categories. Because we have well-defined directed hypotheses about the linear contrasts for the effect of sensory signals and the effect of predictive signals, we used one-tailed tests. Two-tailed tests were used for all the other reported statistics.

### Results

#### Task Performance and Number of Indentations

On average, participant accuracy was approximately 92%. Individual values ranged between 81% and 98%. Performance was significantly higher when predictive signals were high (blocked condition: 93.2%) than when predictive signals were low (random condition: 90.8%), *t*_(15)_ = 3.17, *p* = 0.006. This performance enhancement in the blocked condition as compared to the random condition was present independent whether participants started their first session with the random, *t*_(7)_ = 2.47, *p* = 0.043, or the blocked condition, *t*_(7)_ = 3.63, *p* = 0.008. Additionally, in a between-participant comparison, the number of indentations was significantly correlated with discrimination performance (*r* = 0.62, *p* = 0.010). Hence, participants who explored the stimulus with more indentations had a better performance on average. Overall, participants performed 6.1 indentations per stimulus pair on average, which was more than the minimum of two indentations that would have been necessary to do the task. The individual average ranged from 2.5 to 14.1 indentations. The average number of indentations was not significantly different between the random (6.3) and the blocked (5.8) Presentation Order, *t*_(15)_ = −1.33, *p* = 0.204.

#### Peak Forces

**Figure 4:**
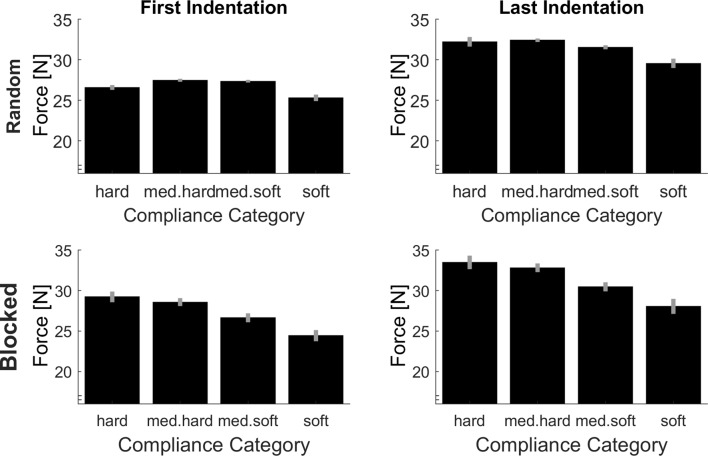
Average peak indentation forces for each condition. Error bars are indicating within-participant standard errors (Morey, 2008).

**Figure 5:**
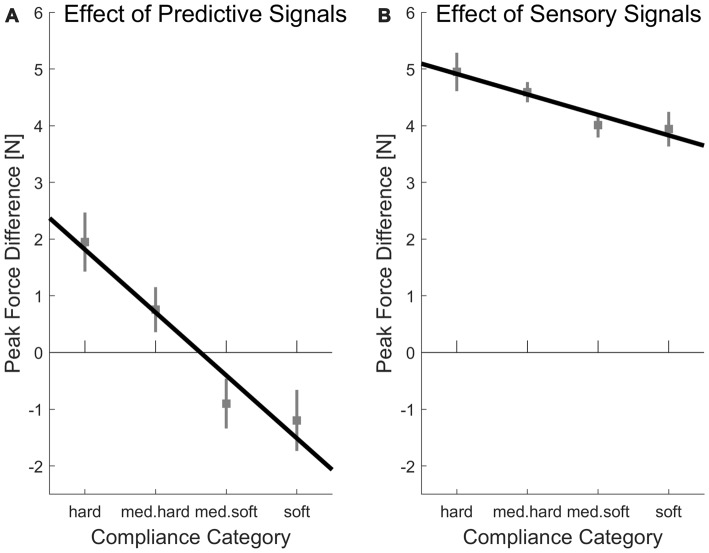
Differences in peak forces produced by **(A)** predictive or **(B)** sensory signals. **(A)** Peak force differences produced by predictive signals were calculated by subtracting values of the random (predictive signals low) conditions from values of the blocked (predictive signals high) conditions. The black line represents a linear contrast of peak force difference on softness category (hard; med. hard; med. soft; soft). **(B)** Peak force differences produced by sensory signals were calculated by subtracting values of the first indentation (sensory signals low) from the values of the last indentation (sensory signals high). Again, the black line represents a linear contrast on softness category. Error bars indicate within-participant standard errors (Morey, 2008).

Average peak forces per experimental case are plotted in Figure 4. On the upper left of this figure, the peak forces in the first indentation (low sensory signals) within the random condition (low predictive signals) are plotted, which constitutes the baseline, for which signals on softness category as much reduced as possible. Importantly and as should be the case, in this baseline, there is no systematic increase of peak forces for softer objects, *t*_(15)_ = 1.64, *p* = 0.122 (linear contrast analysis., two-tailed). Therefore, the difference values which we calculate in the following indicate meaningful adjustments. In other words, higher difference values in indicate that higher absolute peak forces were used. First, we computed linear contrast analyses of the effects of predictive signals (differences in peak force between blocked/high predictive signals and random/low predictive signals condition, see Figure 5A). For the linear contrast, the linear combination of mean values for each softness category was tested against “0” with a one-sided *t*-test. As expected, there was a significant linear contrast over the Softness Categories for one-sided testing, *t*_(15)_ = 3.00, *p* = 0.005, indicating that participants strived to systematically use lower peak forces for softer objects and higher peak forces for harder objects, when softness could be predicted. We then calculated linear contrast analyses based on the effects of sensory signals (differences in peak force between last indentation/high sensory signals and first indentation/low sensory signals, see Figure 5B). Here, the expected linear combination of mean values over the Softness Categories was not statistically significant and showed only a trend in the predicted direction, *t*_(15)_ = 1.24, *p* = 0.088, one-sided.

However, we performed an additional analysis of the movement adjustments based on sensory signals. Similarly to Saig et al. (2012), we calculated coefficients of variation in peak force (standard deviation normalized by the mean) for the first and the last indentation. In agreement with Saig et al. (2012), we found a significant decrease in the coefficient of variation, i.e., a convergence of movement parameters to steady values, when we compared peak forces in the first and the last indentations (*t*_(15)_ = 1.94 *p* = 0.036, one-sided). That is, although there is only a trend for adjustment in peak forces based on sensory signals, the convergence to steady movement parameters might indicate that sensory signals had at least some effect on movement control.

### Discussion Experiment 1

We investigated the influence of predictive and sensory signals on the control of peak forces during softness exploration. We found systematic influences of predictive signals. Participants strived to systematically use lower peak forces for softer objects and higher peak forces for harder objects, when softness could be predicted. This result is in agreement with the existing literature on active movement control (e.g., Johansson and Westling, 1988; Wing and Lederman, 1998; Kaim and Drewing, 2011). Based on the finding that using higher peak forces for hard stimuli enhances discrimination performance (Kaim and Drewing, 2011), we can conclude that the observed movement adjustments optimize movements. We were additionally able to show that the effect of predictive signals is systematic in that it depends on the softness category in a linear fashion.

The expected influence of sensory signals was not significant. In the present study, we observed only a tendency to use lower forces for softer objects based on sensory signals. The follow-up question is, how can we explain the finding that predictive signals had a clear influence on motor control, whereas the relevance of sensory signals was not evident? First, we can consider possible difference in the measurement of the effect of predictive and sensory signals. The effect of sensory signals was measured within one trial (last indentation vs. first indentation) while the effect of predictive signals was measured between sessions (blocked condition vs. random condition). Potentially when comparing force measurements between separate sessions estimates of an effect are likely to be noisier than when comparing two force measurements within one trial of a session. However, this would predict a higher power to detect effects of sensory signals as compared to effects of predictive signals, whereas we found a significant effect of predictive but not sensory signals. Thus, these methodological considerations cannot explain the difference between sensory and predictive signals. However, the literature on motor control might provide an explanation. The most prominent theory of motor control states that our motor system functions like an optimal controller. In the idea of an optimal controller it is suggested that the system uses all available signal sources, but weights those signals that are more reliable more heavily (e.g., Saunders and Knill, 2004). This is the same principle that theories of optimal signal integration describe for perception (Ernst and Banks, 2002). In order for one source of signals to show a major effect on movement control, it has to be sufficiently reliable to be weighted heavily in the computation. Consequently, it may be that in our experiment, the reliability of sensory information was not sufficient to warrant a significant effect on movement control. In active touch, sensory information gains reliability with extension of exploration; the more movements performed, the more sensory signals are generated, and the more reliable the sensory information overall will be (Lezkan and Drewing, 2018). Therefore, it seems possible that our participants did not explore with the necessary extension to generate sufficient sensory signals.

The fact that people do not necessarily use a maximum number of movements for a task was previously explained in movement control literature (Todorov and Jordan, 2002; Todorov, 2004) by the additional energetic effort every extra movement costs. It was suggested that movement costs (in terms of effort) are counterbalanced with the rewarding nature of the performed movement. This means that higher energetic costs should only be spent if they increase the reward. Corroborating these notions, research from visual perception indicates that the expectation of reward impacts eye movements. When rewarded, saccades (especially longer saccades) had higher peak velocities and shorter latencies in monkeys and humans (Takikawa et al., 2002; Xu-Wilson et al., 2009; Schütz et al., 2012). This is evidence for a link between higher energetic effort and expected reward in visual perception. We speculate that similarly our participants might not have gathered sufficient sensory signals because they were not expecting higher reward for high perceptual performance. In Experiment 2, we investigate the influence of motivational factors. In the present case, we refer to the rewarding value of a task as the motivation to perform it (Beckmann and Heckhausen, 2006). We investigate whether motivation influences the effort spent for exploration movements in haptic perception. In particular, we assume that high reward (yielding high motivation) increases the gathering of sensory signals and the online adjustment of movement.

## Experiment 2

We manipulated motivation via the possibility to win money with each correct response in half of the experiment (motivation part) and pretending that the system does not work in the other half (demotivation part). Participants performed a 2AFC softness discrimination task among stimulus pairs from either the soft (0.61–0.73 mm/N) or the hard (0.15–0.16 mm/N) category. With higher motivation, we expected more pronounced adjustments of peak force based on sensory signals. Thus, motivation should moderate the effect of sensory signals on movement adjustments. Specifically, we expect a statistically significant linear contrast when calculating the differences between the two motivation conditions in the effect of sensory signals (i.e., the peak force differences between the first and the last indentation) for each softness category. Please note, in the 2 × 2 design the expected linear contrast is equivalent to the interaction effect in a standard ANOVA.

### Materials and Methods

#### Participants

Sixteen participants (10 female; mean age: 25.6 years, range: 20–32 years) entered in the final sample based on a successful manipulation of motivation. For 14 other people, who filled in the questionnaire, we were not able to manipulate motivation as intended. The *a priori* defined inclusion criterion was that motivational values (i.e., the points achieved in the motivational questionnaire) in the motivational part were higher than motivational values in the demotivation part. Therefore, participants, who did not meet this criterion, were not part of the main analysis. Participants were compensated by 24–32 € (29 € on average). The compensation was calculated from a fixed value (12 €) for the demotivation part of the experiment, plus the monetary equivalent of the achieved points in the motivation part with a fixed bonus (in total 12–20 €, 17 € on average).

#### Apparatus and Stimuli

The apparatus and setup were identical to Experiment 1. In each softness category, the standard was paired with one of two comparisons (“soft”: 0.61, 0.67, 0.73 mm/N; “hard”: 0.152, 0.156, 0.162 mm/N). Compliance differences of the two comparison and the standard were either easier or more difficult discriminate. We chose stimuli in adaptive piloting procedure (duration: 30–45 min per participant). Thirteen participants (who were not part of either of the main experiments) explored stimulus pairs of either both or one of the softness categories. We changed the stimuli of a pair across participants until at least two participants showed a performance of approximately 90% for one pair and 80% for the other pair of the same softness category.

#### Design and Procedure

Motivation (motivation vs. demotivation) and Softness category (hard vs. soft) were manipulated as within-participant variables. The manipulation of motivation restricted us to a single-session design. Given the single-session design we did not manipulate presentation order as a within-participant variable, because it was not the focus of this experiment to look for interactions of predictive signals with motivation. The presentation order was approximately balanced between the participants in the final sample (nine blocked, seven random), but not further analyzed, because this design does not provide sufficient statistical power for these analyses. As in Experiment 1, a 2AFC softness discrimination task was used. Visual cues were similar to Experiment 1, except for an additional screen to inform participants about gaining monetary rewards.

The experiment consisted of one session with four blocks of 112 trials (448 trials in total), and breaks were given between blocks. The total experiment took about 3 h. In each block, the eight stimulus pair combinations were repeated 14 times. Stimulus pair combinations were defined by the compliances of the stimulus pair and the positioning (standard left vs. right). Two successive blocks constituted the motivation part and two other successive blocks constituted the demotivation part. We approximately balanced the order of conditions between the participants in the final sample (nine started with the motivation condition, seven started with the motivation condition). All participants were instructed to consider the experiment as a game, in which they can gain points. Before the start of the exploration, a screen indicated how many points (50 or 100) could be gained with a correct response (randomly associated to half of the trials each). Whenever the participant accumulated 1,000 points, an additional euro was gained. Thus, based on the pilot data, we estimated that participants accrued 1 € every 18 trials. Visual and auditory feedback was given one to three trials after gaining an additional euro. This gaining rule was only true for half of the experiment (two subsequent blocks; motivation part). For the other half of the experiment, we pretended that the reward system stopped working and we had to reimburse participants with the conventional payment (demotivation part). We induced demotivation in this way, because a not working system implies that a reward will be expected but not given. Losing the expected reward should be weighted more than not being rewarded (see Crespi, 1942; Tversky and Kahneman, 1974) and we wanted to maximize the difference between the two within-participant motivation conditions as much as possible. This “error” was presented by the system displaying a zero for the points in each trial. For participants who started with the demotivation part, we pretended that the only person able to fix this “error” was not reachable at first. However, after the first experimental part (demotivation), this person came back and was able to fix the “problem” so that the second half was conducted with the possibility to win money (motivational part). For the other half of participants, the experiment worked as instructed in the first half of the session. After the second break, the “error” appeared. Again, no one who could fix it was reachable and the second part of the experiment did not allow participants to gain points (demotivation).

#### Motivational Questionnaire

In order to ensure the manipulation, a motivational questionnaire was given to participants after each experimental half. The questionnaire was constructed by adapting two questionnaires that access task motivation and adding four items that capture the social desirability bias (used for distraction and individual correction (see below)). The basis for the motivational questionnaire was the following two surveys: potsdamer Motivationsinventar (“PMI,” Rheinberg and Wendland, 2002), originally used to measure task-specific motivation values in a school environment, and the PANAVA scale (PA, positive activation; NA, negative activation; VA, valence) which measures one’s mental state while performing a task (Schallberger, 2000).

To measure social desirability, we chose four items from the German SDS-E scale of social desirability (items 7, 8, 17, 22; Lück and Timaeus, 2014), which would not stand out if added to the PMI scale. Our adapted PMI scale included all 10 items from the original PMI, in which we only modified task-specific expressions, like “mathematical task” into “this task.” The responses were given on Likert-type items, which could be rated from “does apply” (1) to “does not apply” (5). Two examples are: “I wish I did not have to perform this task” and “Performing this task has positive effects on my mood.” The PANAVA scale asks how the participant felt “directly before starting a trial.” Assessments were done on a seven-step scale between two adjectives, representing opposite poles of one dimension. Four items captured positive activation (awake vs. tired; full of energy vs. shiftless; energetic vs. inert; excited vs. bored); two items measured valence (happy vs. unhappy; satisfied vs. unsatisfied). These six items are positively related with motivation. The other four items measured negative activation (relaxed vs. stressed; good-humored vs. upset; calm vs. nervous; carefree vs. worried), which is negatively related to motivation. The questionnaires were rated after each experimental part (motivation vs. demotivation). For each item’s response, the associated values were read out as points. For each questionnaire, a range between the minimum and maximum sum of points was defined (PMI: min. 10, max. 50; PANAVA: min. −22, max. 38, note: NA only contributes negative points) and transformed in percentages between the minimum (0%) and maximum (100%) sum of points. An uncorrected motivation score was calculated by averaging these two motivational values from the two scales for each experimental part. Additionally, to improve the validity, individual motivation scores were corrected by the social desirability score. As a corrected motivation score, we used the residuals of a linear regression of uncorrected motivation scores on social desirability, which is common practice in several scales as the MMPI (Lubin, 1957; Paulhus, 1981).

#### Data Analysis

We estimated the exhibited energetic effort of the exploration in one trial by the sum of peak forces over all indentations performed in this trial. Additionally, we calculated individual peak forces per condition and the effects of sensory signals on peak force, as described in Experiment 1. Because we have directed hypotheses about the effect of motivation on the task performance, the energetic effort and the linear contrast produced by sensory signals we used one-tailed tests for these analyses.

### Results

#### Questionnaire

On average, participants in the final sample reported 39.5% (SD = 12.5%) from the maximal points they could achieve in the motivational questionnaire in the demotivation part and 49.1% (SD = 7.7%) in the motivation part. This difference between the motivation conditions was statistically significant, *t*_(15)_ = −4.89, *p* < 0.001. Additionally, differences in motivational values between the motivation conditions in the subgroup that started with the motivation condition were not significantly different from the difference values in the other subgroup, *t*_(14)_ = 1.61, *p* = 0.320. The data from participants, that we excluded based on the questionnaire are fully consistent with our hypothesis: as it is to expect from the not successful manipulation, their data showed no differences in performance, effort or movement adjustments between the conditions.

#### Task Performance and Energetic Effort

**Figure 6:**
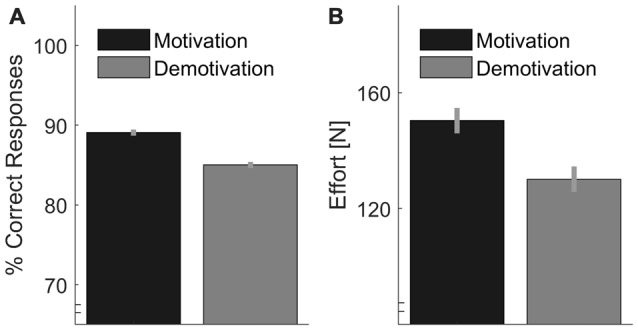
**(A)** Performance, as measured by the percentage of correct responses, plotted separately for motivation vs. demotivation. **(B)** Estimate of effort per trial based on the sum of peak forces from all indentations of one trial. Error bars indicate within-participant standard errors (Morey, 2008).

We calculated individual percentages of correct answers (Figure 6A). We analyzed whether Motivation significantly increased performance with a one-sided *t*-test (after rationalized arcsine transformations of the individual proportional data). We found a significant effect of Motivation, *t*_(15)_ = 4.43, *p* < 0.001 with 88.9% (SD = 5.7%) correct answers in the Motivation condition vs. 85.3% (SD = 7.6%) in the Demotivation condition. Further, values of energetic effort (Figure 6B) were tested in the same way. As expected, we found a significant effect of Motivation in one-sided testing, *t*_(15)_ = 2.06, *p* = 0.029.

#### Peak Forces and Motivational Effects on Adjustments to Sensory Signals

**Figure 7:**
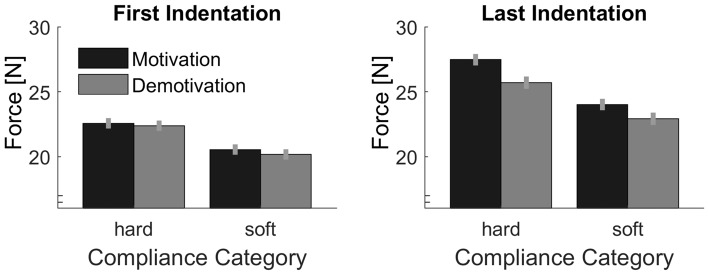
Average peak indentation forces for each condition. Gray bars represent the demotivation part and black bars the motivation part. Error bars indicate within-participant standard errors (Morey, 2008).

**Figure 8:**
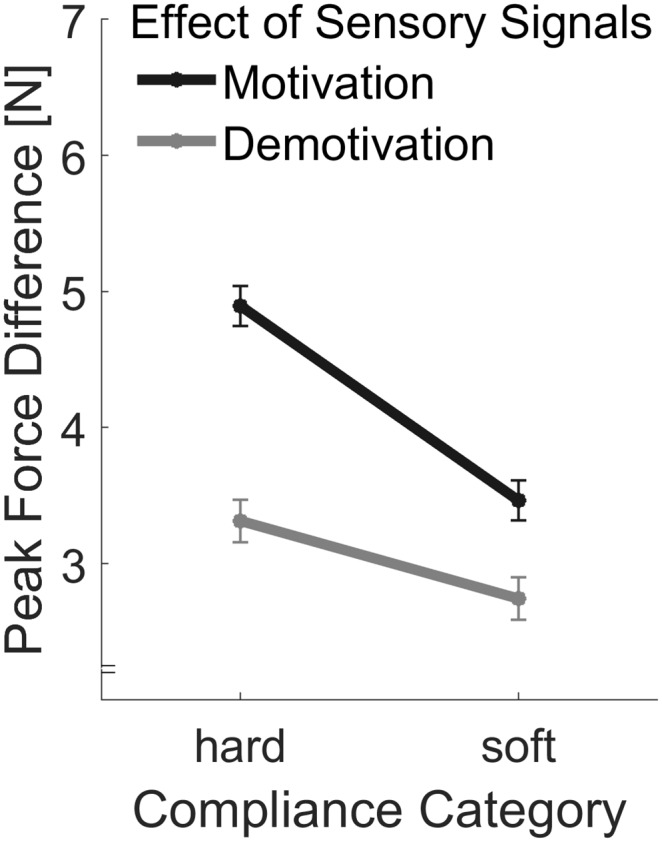
Differences in peak forces produced by sensory signals. The peak force differences were calculated by subtracting values of the first indentation from the values of the last indentation. Error bars indicate within-participant standard errors (Morey, 2008).

Peak forces are plotted in Figure 7. As in Experiment 1, we calculated the difference between the first and the last peak forces in each trial in order to assess effects of sensory signals (Figure 8). First we computed, the linear contrast analyses, of mean force difference values over the Softness Categories with a one-sided *t*-test. The linear contrast analyses over both Motivation conditions, which represents the overall adjustments to sensory signals, revealed a statistically significant effect, *t*_(15)_ = 2.71, *p* = 0.016. More importantly, we calculated the linear contrast on the differences in mean values between the two Motivation conditions, which revealed a significant interaction between Motivation and the linear contrast on Softness Category in the one-sided test, *t*_(15)_ = 2.25, *p* = 0.020. This result supports our hypothesis, that effects of sensory signals were higher in the motivation part. That is to say, peak force adjustments based on sensory signals were higher with higher motivation.

In order to test whether adjusted movements based on sensory signals were moderated by the experimental half, we performed the same analyses as previously but with experimental half (instead of motivation) as the moderator. Therefore, we calculated the linear combination of the differences in force difference values between the two experimental halfs and tested it with a two-sided *t*-test against “0.” The experimental half, was not a significant moderator of the effect of sensory signals, *t*_(15)_ = 1.24, *p* = 0.235.

### Discussion Experiment 2

The motivational manipulation influenced the exploration process as expected: when motivated, participants adjusted movements based on sensory signals more profoundly as compared to when being demotivated. Additionally, participants spent more energetic effort for the exploration and performed better when motivated.

Our participants started the experiment with either the motivation or the demotivation condition which we approximately counterbalanced (9–7). One could ask whether this slight disproportion in favor of the participants who started in the motivation condition might have caused the effect. That is to say, did the experimental half influence movement adjustment instead of the motivation condition? As we did not find a significant moderation of the effect of sensory signals by the experimental half, our results speak against the experimental half as a possible confound in our data set.

An increase of the effect of sensory signals, as produced in the motivation condition, could be either due to the availability of more sensory signals or to a higher weighting of sensory signals in motor control. The present results show that participants not only improve motor adjustment, but also show better perceptual performance and generate more sensory signals when being motivated. Therefore, in our case, changes in online adjustment of movements are more parsimoniously explained by the acquisition of more sensory signals. Although, the acquisition of additional sensory signals seems to rule out the up-regulation as an alternative explanation in our experiment, previous literature has reported conditions under which sensory signals gain or lose influence on motor adjustment without any change in their availability (e.g., Jakobson and Goodale, 1991; Knill et al., 2011). For instance, Knill et al. (2011) demonstrated that, after a perturbation of the visual feedback, participants corrected their pointing movements more if the accuracy demands of the task were greater.

## General Discussion

We investigated whether peak indentation forces are adjusted based on predictive or sensory signals to softness category, in natural exploration. Participants systematically used higher peak force for harder objects when they were informed about the softness by predictive signals. Interestingly, self-generated sensory signals in Experiment 1 had a less clear impact on movement adjustments (if any) than predictive signals. We reasoned this to be due to a lack of motivation to generate sufficient sensory signals. Thus, in Experiment 2, we manipulated the motivation to do the task in order to modulate the effect of sensory signals. When participants were motivated, they adjusted their peak forces significantly to the sensed softness. We associated this with the generation of additional sensory signals, because participants also spent more energetic effort for exploration when motivated. This was additionally indicated by an improved perceptual performance. Taken together, this study provides evidence that softness exploration constitutes a closed sensorimotor loop, where prediction, sensation and motivation are relevant determinants of movement control.

In our experiments, we showed that when participants adjusted their peak force more precisely to the object they also generated more sensory signals. In previous work, we additionally showed that perception gets more precise with more generated sensory signals (Lezkan and Drewing, 2018; Metzger et al., 2018). In sum, accumulating sensory signals seems to improve the precision of movement and perception. Therefore, we propose that in natural softness exploration, strong links exist between sensory signals and following movements. We believe that the softness estimates used for motor control are similar to softness estimates used for perception. However, there is a long-standing debate on whether sensory signals are used in the same manner when being processed for action vs. for perception (Goodale and Milner, 1992; Smeets and Brenner, 2006). Interestingly, Leib et al. (2015) described for a task, similar to our own, a dissociation between the use of sensory signals for movement control and for perception. In their experiment, participants explored the stiffness of virtual elastic force fields using a tool. When the force feedback was delayed, participants underestimated the stiffness, meaning that perception did not discount the temporal delay. In contrast, their grip forces, with which they were holding the tool, were adjusted to the force feedback delay. However, in that study, not only the use of sensory signals differed between perception and action, but also the tasks for which the sensory signals were used: the motor task involved keeping a stable grip, while the perceptual task required estimating the stiffness of an object. Thus, the motor control required information about the time course of feedback force in order to program grip forces that warrant a stable grip. In contrast, for the perceptual task force feedback and position feedback had to be combined into an estimate of stiffness. Thus, the dissociation observed in Leib et al. (2015) might be caused by the differences between the perceptual and the motor task, rather than a differential use of the same signals (see Smeets and Brenner, 2006). In the present study, sensory signals have been used for the same basic task, namely to derive a softness estimate. Future experiments are required to test our assumption that softness estimates used for perception and for motor adjustment are indeed highly linked.

Based on our observations, we can summarize several observations about movement control in natural exploration: exploratory movements seem to be executed with the aim of enhancing performance. When possible, our motor system uses predictions to lower movement costs. Additional exploration movements are performed when perceptual performance is not yet at the target level. However, this active sensory gathering is moderated by motivation. Thus, our results may be taken to suggest that the aim of motor control is to change the internal state to be more rewarding. This can happen internally by a better perceptual representation or externally by reward. If so, motivation could be understood as a driving mechanism in the motor control system. So far, this is rather a hypothesis which we derive from our results and which should be investigated in future.

In our study we tried to systematically clarify the mechanisms underlying movement control for softness exploration. However, more research is needed in order to understand whether the described mechanisms can be generalized to other natural exploration behaviors. Understanding general mechanisms of movement control in natural exploration might also be useful to help in cases, when these mechanisms do not work. Several links between abnormalities in the sensorimotor mechanisms and psychological dysfunctions have been suggested. In a recent study, Mosconi et al. (2015) reported that patients with an autism spectrum disorder show impairments in feedforward as well as in feedback processes of sensorimotor control. Additionally, Shadmehr et al. (2010) discussed the relation between diseases of the reward system, such as Parkinson’s disease or schizophrenia and movement control. The authors suggest, similar to our conclusions, that rewards are driving motor signals and see these diseases in the context of a discounting of rewards, which can be achieved with motor commands. Our study offers a first step in understanding the role of motivation in motor control for natural exploration movements. Further systematic research about factors influencing motor control may not only help to understand natural exploration behavior, but also diseases of the movement system.

Apart from the investigated factors, which seem to be indispensable for a functioning motor control, our data allows to speculate which additional variables may influence motor control in natural softness exploration. One interesting observation is that participants did not use a fixed order of indentations for the two stimuli. In the last indentation before giving a response, participants touched disproportionally more often the stimulus, which they were about to choose (Experiment 1: 75.74%; Experiment 2: 72.68%). Similar behavior of fixating the object right before choosing it was also reported for vision (Krajbich et al., 2010; Manohar and Husain, 2013). In the haptic modality, Mitsuda and Yoshioka (2015) described that participants tended to sample last the object they reported to be more preferable to the other object. This behavior could reflect attention or decision making processes. One possibility is that participants perform the last movement to reaffirm their choice based on the sensory signals gathered up to that point.

Additionally, we observed an unexpected general effect of the exploration moment (last vs. first indentation) on peak forces. In the last indentation, peak forces increased in comparison to the first indentation (Experiment 1: *t*_(15)_ = −3.81, *p* = 0.002; Experiment 2: *t*_(15)_ = −3.99, *p* = 0.001). This increase happened gradually, given that it was also reflected in the middle indentation. One possible explanation is that through perceptual adaptation to force (see Vogels et al., 2001), the softness sensitivity diminished. Every indentation is associated with a force profile on the finger tip, which varies over time and space. The adaptation to pressure was one of the first characteristics described for mechanoreceptors (Zigler, 1932; Nafe and Wagoner, 1941; Johnson, 2001). Adaptations on a neural basis are reflected in changed perception (Cohen and Vierck, 1993). Consequently, repeated indenting within static contact with the object might lead to diminished neural responses, and thus, reduced sensation during softness exploration. On the other hand, increases in contact force between finger and stimulus were previously observed to scale population responses upwards (Goodwin et al., 1995). Thus, increasing indentation force might be a way to counteract declining neural response due to adaptation. That is to say, increasing indentation force could be a reasonable strategy to counteract negative effects of such perceptual adaptation. However, this is mere speculation at this point and further research is needed. Taken together, we assume that although prediction, sensation and motivation have a high impact on movement control in natural exploration, there are likely further impact factors, including bottom-up factors like adaptation, or top-down factors, like decision-making. In order to build a model of natural motor control, several of those factors have to be further investigated.

## Conclusion

This study provides new and important insights in movement control within unconstrained haptic softness exploration. Participants applied systematically lower forces in the exploration of softer objects when the softness category was predictable, or previously experienced within the exploration of this stimulus. Based on this finding, softness exploration can be understood as a sensorimotor control loop containing a feedforward process based on predictive signals, and a feedback process, based on sensory signals. The roles of the feedforward and feedback processes seem to change during the exploration. While the existence of a feedforward process influences movement control during the entire exploration process, the feedback process gains importance as more sensory feedback is gathered over time. Our findings highlight the role of motivation as a moderator of feedback processes. Increased motivation led to an increase in motor adjustments based on sensory signals. Overall, such a system seems to aim for the most effective way to perform a task. Movements are chosen as appropriately as possible at a given point in time. Energetic effort of the movements is kept low in order to achieve an aimed performance.

## Author Contributions

AL and KD designed the experiment. AL collected and analyzed the data. AL, AM and KD wrote the article.

## Conflict of Interest Statement

The authors declare that the research was conducted in the absence of any commercial or financial relationships that could be construed as a potential conflict of interest.
